# A Diverse Community To Study Communities: Integration of Experiments and Mathematical Models To Study Microbial Consortia

**DOI:** 10.1128/JB.00865-16

**Published:** 2017-07-11

**Authors:** Antonella Succurro, Fiona Wanjiku Moejes, Oliver Ebenhöh

**Affiliations:** a Botanical Institute, University of Cologne, Cologne, Germany; b Cluster of Excellence on Plant Sciences (CEPLAS), Düsseldorf, Germany; c Institute for Quantitative and Theoretical Biology, Heinrich-Heine-Universität, Düsseldorf, Germany; d Bantry Marine Research Station, Gearhies, Bantry, Co. Cork, Ireland; Geisel School of Medicine at Dartmouth

**Keywords:** interdisciplinary approaches, marine ecosystems, mathematical modeling, microbial communities

## Abstract

The last few years have seen the advancement of high-throughput experimental techniques that have produced an extraordinary amount of data. Bioinformatics and statistical analyses have become instrumental to interpreting the information coming from, e.g., sequencing data and often motivate further targeted experiments. The broad discipline of “computational biology” extends far beyond the well-established field of bioinformatics, but it is our impression that more theoretical methods such as the use of mathematical models are not yet as well integrated into the research studying microbial interactions. The empirical complexity of microbial communities presents challenges that are difficult to address with *in vivo*/*in vitro* approaches alone, and with microbiology developing from a qualitative to a quantitative science, we see stronger opportunities arising for interdisciplinary projects integrating theoretical approaches with experiments. Indeed, the addition of *in silico* experiments, i.e., computational simulations, has a discovery potential that is, unfortunately, still largely underutilized and unrecognized by the scientific community. This minireview provides an overview of mathematical models of natural ecosystems and emphasizes that one critical point in the development of a theoretical description of a microbial community is the choice of problem scale. Since this choice is mostly dictated by the biological question to be addressed, in order to employ theoretical models fully and successfully it is vital to implement an interdisciplinary view at the conceptual stages of the experimental design.

## INTRODUCTION

Theoretical biology is a very broad field where mathematical and physical concepts are used to describe biological systems. Computational models are being widely applied in the investigation of biological phenomena, and their potential goes well beyond simply reproducing and validating experimental data. To achieve predictive power, however, a theoretical model needs to be fully integrated into the experimental design. Indeed, modeling techniques are very diverse and their efficacy is highly specific to the problem scale and the objective of the study. The selection of the most appropriate method for the question at hand depends not only on the intuition and experience of the modeler but also on the type and quality of the data available (1). This is why it is crucial that modelers and experimentalists come together at the conceptual stages of a project to jointly plan experiments, measurements, and data management. An experimental protocol design that ignores the modeling aspect is set up to obtain data that would most likely be suboptimal for modeling.

The research interest in microbial communities is gaining momentum, and we think that now is the time for setting new standards in the scientific methods for understanding and potentially engineering such complex systems. It is our impression that rapid technological advances in experimental techniques providing high-throughput data are not accompanied by proportional advances in the theoretical methods to interpret them systematically. Interdisciplinary studies of microbial consortia are becoming more common (2) but are still exceptions rather than the norm, especially if compared to the fields of ecology (3) and metabolism (4), where mathematics has since long been integrated. In this minireview, we want to provide a glimpse of the broad ranges of available mathematical models. We use the example of marine ecosystems with their wide variation of complexity scales to highlight how experiments and theory can be successfully paired, with the goal of motivating scientists to engage in a multidisciplinary approach for understanding microbial interactions.

## MATHEMATICAL MODELS FROM THE OCEAN TO THE PHYCOSPHERE

Classical terminology defines an ecosystem as a community of living organisms interacting with one another and with the physical environment. Covering approximately 70% of Earth's surface and contributing to one-half of global primary production (5, 6), the ocean is the largest biome on our planet and provides illustrative examples of ecosystems exhibiting spatial and temporal heterogeneity. With an estimated 10^4^ to 10^6^ cells per milliliter (7), marine microbes make up the vast majority of oceanic biomass but have historically been ignored by oceanographers (8). We have only recently begun to understand their importance in fundamental phenomena such as biogeochemical cycling and primary productivity which play vital roles in the ability of animals and plants to exist and thrive on Earth. Members of the plankton ecosystem (viruses, bacteria, archaea, protists, and metazoans) have recently been systematically collected in their natural habitats (9) to explore ecosystem dynamics at the ocean scale. However, much is still unknown about how these microorganisms interact with one another (Table 1 provides examples of marine microbial interactions).

**TABLE 1:** Types of marine microbial interactions

Relationship	Diagram	Example	References
Mutualism	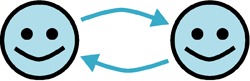	A Sulfitobacter species promoted cell division of a coastal diatom, Pseudo-nitzschia multiseries, via secretion of the auxin phytohormone indole-3-acetic acid synthesized by the bacterium using diatom-secreted tryptophan	10, 11
Competition	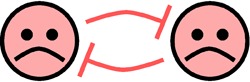	Competition for “free” orthophosphates in the predominantly nutrient-limited marine biome; marine bacteria are better competitors for phosphorus than eukaryotic algae at low ambient nutrient concentrations	12–15
Parasitism	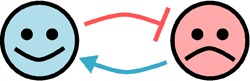	Lytic viral infection of other single-celled organisms by attachment of virus to a host cell and injection of its nucleic acid into the cell, directing the host to produce numerous progeny viruses; these are released by fatal bursting of the cell, allowing the cycle to begin again	16–18
Predation	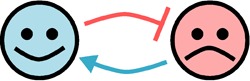	Ciliated bacteriovores in marine environments such as aloricate oligotrichous ciliates graze on bacteria	19–21
Commensalism	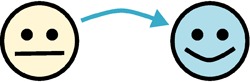	Certain bacteria found in the algal sheath where they look for carbon and shelter with no effect on the algal host	22, 23
Amensalism	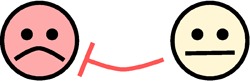	Marine bacteria such as Kordia algicida and Pseudoalteromonas sp. strain A28 can secrete enzymes capable of lysing diatoms	24–26
Neutralism	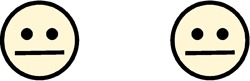	A debated phenomenon, often suggested to rarely exist in natural communities but possibly occurring in ecosystem structures where two species are too far apart spatially; industrial examples exist; e.g., the population densities of a Lactobacillus sp. and a Streptococcus sp. grown in continuous culture were observed to be similar in mixed cultures and in individual cultures	27–29

A wide range of theoretical modeling techniques has been developed to address different temporal and spatial scales. The flexibility of a model, combined with rigorous mathematical thinking, allows the translation of working hypotheses into reproducible *in silico* experiments. In this way, mechanistic insights into fundamental processes as diverse as evolution and metabolism can be gained. The relatively low cost of generating copious amounts of simulated data enables testing of an assortment of hypotheses with a freedom not achievable with *in vivo*/*in vitro* experiments. With this freedom, however, comes the risk of deviating from biologically meaningful results, and it is therefore necessary to plan a continuous and reciprocal integration of simulations and experiments. In this section, we provide a short overview of mathematical methods used to model microbial communities with a focus on ocean-wide systems, biofilms, and the phycosphere. The reader interested in more extended reviews of mathematical modeling of microbes is referred to references 27 and 30.

## DIFFERENT UNITS FOR DIFFERENT QUESTIONS

A general rule when developing a mathematical model is that one should keep it *“*as simple as it can be, but not simpler*”* (words attributed to Albert Einstein by the writer and composer Roger Sessions in 1950). As simple as it sounds, this remains one of the main challenges in modeling, as a “combinatorial explosion” of the number of variables and parameters can easily occur. This is particularly true in natural microbial ecosystems, where diversity levels are high and countless cell-to-cell and cell-to-environment interactions can be considered. Ranging from meters to kilometers in size, large marine ecosystems include relatively permanent systems such as coral reefs and salt marshes but also temporary phenomena such as algal blooms (31). Spanning meters to millimeters, marine biofilms are found on mineral macrostructures (e.g., rocks) and man-made infrastructures (e.g., ships) but also on small-scale organic and inorganic matter in oceans (“marine snow”) (87). Biofilms are the result of growth and aggregation of microbial organisms and their exudates, particularly extracellular polymeric substances, on a surface (32, 33). Zooming in to the micro- and nanometer scale, cell-to-cell relationships include interactions that occur in the phycosphere, a term coined by Bell and Mitchell in 1972 (34) to denote the region extending outward from the microalgal cell in which bacterial growth is stimulated by extracellular products of the eukaryote.

**FIG 1:**
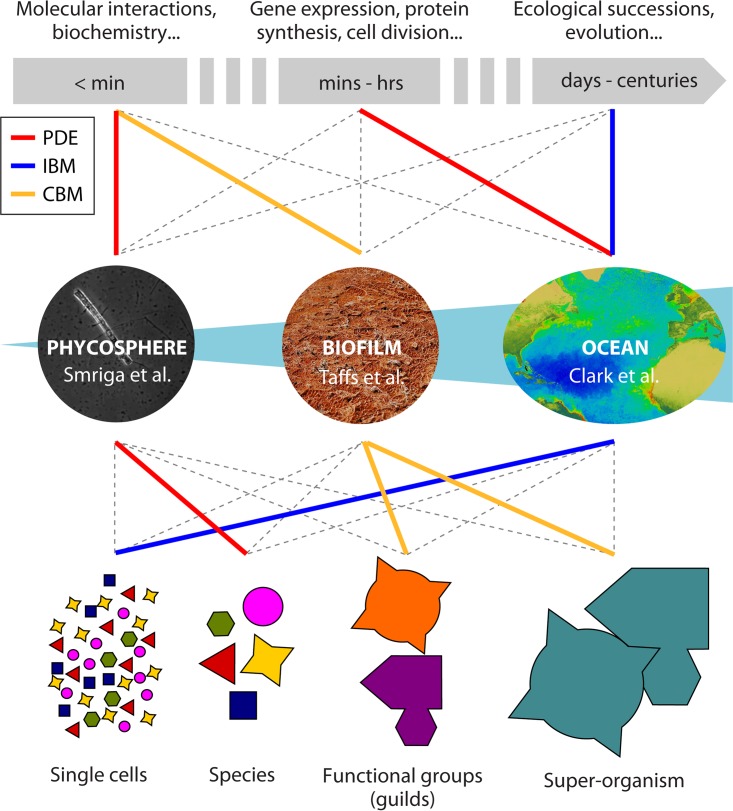
Schematic representation of different choices of temporal (top) and spatial (bottom) scales for the same ecosystem (center). PDE, IBM, and CBM methods are represented with red, blue, and yellow lines, respectively. Smriga et al. (80) used PDE to model a phycosphere community at the species level (picture from Smriga et al. [80]). Taffs et al. (74) used CBM to model a biofilm at the guild and superorganism levels (the picture of thermophilic bacteria in Mickey Hot Springs, Oregon, is from https://en.wikipedia.org [contributed by Amateria1121]). Clark et al. (53) used IBM plus PDE to model an ocean system at the single-cell level (the ocean picture is from the SeaWiFS instrument [https://svs.gsfc.nasa.gov/]).

Different levels of abstraction can be defined, determined by the community dynamics at the core of the research question. Figure 1 illustrates examples of the scales at which systems of the marine biome can be investigated independently of their actual size. Starting from single-cell resolution, we can first average individuals as a whole population (species) and then further reduce the complexity by considering functional groups (also called guilds) of species performing the same role in the ecosystem and finally define a single superorganism interacting with the environment. The choice of problem scaling is determined by the research question and is not directly related to the physical size of the biological system. For example, effective models can be built of oceanic ecosystems at the cell level and of a bacterial culture as a single superorganism. A similar argument holds for temporal scales, with the difference being that the time scale is an intrinsic characteristic of the phenomenon being addressed. Indeed, the same ecosystem can be investigated at the biochemical level or in an evolutionary perspective, a choice that will set the time constants at the order of fractions of seconds or centuries, respectively.

## POPULATION MODELS

Population-scale models were among the earliest applications of mathematical models, dating back to 1838 when the mathematician Verhulst developed the equation that describes logistic growth (35). Almost a century later, Lotka (36) and Volterra (37) independently described the oscillatory behavior of multispecies populations with a system of ordinary differential equations (ODEs), developing one of the first ecosystem models. ODEs describe the temporal change of a variable (e.g., a population) as a function of other dynamic variables as well as of parameters that define external (e.g., environmental) factors. One variable may positively (if it causes an increase) or negatively (if it causes a reduction) influence another variable. ODEs do not include spatial inhomogeneities, but these can be described by partial differential equations (PDEs). Due to their versatility and ease of implementation, differential equations (38) are among the tools most widely used in kinetic modeling methods and have been applied to understand phenomena in fields such as economics, hydrodynamics, and biochemistry.

Table 1 illustrates how microbial interactions can be classified into positive, negative, and neutral actions. Deciphering the interplay between the microbial population and the physicochemical environment is a critical step to further understanding and predicting phenomena related to ecological succession (39). In 1946, Gordon Arthur Riley was arguably the first to describe the phytoplankton annual cycle with mathematical models based on concentrations of dissolved nutrients, solar radiation, and other environmental factors (40, 41). Recent advancements in network inference techniques have allowed the reconstruction of interaction networks from co-occurrence patterns in time series of metagenomic sequencing data (42). Such networks are used to build generalized Lotka-Volterra (gLV) models, where each species is parameterized with a growth rate and a strength of interaction with other community members (43).

Hoffmann et al. (44) developed a gLV model to describe the dynamics of marine phages preying on bacteria (45). They tested different population distributions to explain metagenomics data from shotgun experiments performed on natural marine phage communities and found a modified gLV model to fit the data best. The equation form of the model is interpreted as a cooperation mechanism for phage predation and predicts that the community follows kill-the-winner dynamics where blooming periods are followed by rapid decay. As was demonstrated in gLV models of gut bacteria communities (46), other effects such as environmental perturbations can be also introduced.

## INDIVIDUAL-BASED MODELS

Conventionally, mathematical models based on differential equations are deterministic and do not capture effects of stochastic processes. Individual-based models (IBMs) (47)—sometimes referred to as “agent-based models”—implement Monte Carlo techniques to introduce the randomness required to model the dynamics of single individuals. In these discrete models, each unit temporally evolves to the next stage according to probabilistic rules, making them ideal for describing the single-cell level. High degrees of complexity can be introduced to account for interaction mechanisms as well as individual variability, parameterized as metabolic states and mutation rates, to name two. IBMs are widely used in diverse disciplines and can be successfully applied to describe different temporal and spatial resolutions (47, 48).

A comprehensive review by Costerton et al. (49) reports that biofilms predominate, numerically and metabolically, in virtually all nutrient-sufficient ecosystems. Understanding the metabolic cooperation dynamics is fundamental, and the bacterial composition (relative abundance and spatial distribution of the species) of a biofilm is mainly determined by three processes that take place within the film: (i) conversion of substrates by bacteria, (ii) volume expansion of biomass, and (iii) transport of substrates by molecular diffusion. Early models of biofilm development used a set of mass balance PDEs (50, 51), and IBMs (adding individual variability factors) and PDEs (describing molecular diffusion) have been extensively combined over the last decade. Within a model, however, different scales have to be carefully integrated in a theoretically justified manner. For example, processes occurring over shorter time scales can be considered to be in a quasi-steady state in comparison to processes occurring over longer time scales. This is the case in the framework proposed by Lardon et al. (52), where solute diffusion and reaction dynamics are considered to be in a quasi-steady state with respect to biomass growth. They investigated individual metabolic switching in response to environmental changes in a community of denitrifying bacteria and identified a tradeoff between cost and response time, explaining the maintenance of different denitrifying strategies in fluctuating environments.

Despite the correlation between increasing system size and computational “cost,” Clark et al. (53) implemented IBMs to model an ocean-scale system. The authors developed a trait-based model where a single agent represents individuals with identical traits. An ocean PDE model discretized into cell grids provides the environmental conditions (temperature, salinity, and diffusion of dissolved nutrients), and agents are mixed between the spatial units. By performing simulations on an evolutionary time scale, it was demonstrated that different environments select for different traits such as cell size in phytoplankton.

## METABOLIC NETWORK MODELS

By correctly assigning metabolic functions to the enzymes encoded in a whole genome, it is possible to obtain a genome-scale metabolic network model (54, 55). This reconstruction is close to becoming an automated process (56, 57), but it still requires careful manual curation thereafter, because incomplete or inaccurate genome annotations can lead to unreliable models (58). Various mathematical methods have been developed to analyze metabolic network models. Network expansion (59) is an error-tolerant qualitative method useful to infer minimal nutrient requirements (60) and to classify organisms by lifestyle based on their metabolic capabilities (61). Christian et al. (62) demonstrated how the range of possible biosynthetic products increases if two or more organisms cooperate on a metabolic level, illustrating the potential of synergistic interactions of organisms (including bacteria). Stoichiometric models (4, 63, 64) are mathematical representations of metabolic networks in the form of a matrix with the stoichiometric coefficients of the reactions present as elements. At the steady state, the requirement of mass balance translates into a mathematical system of equations whose solutions represent feasible metabolic fluxes. Because the solution is not unique, constraint-based models (CBMs) (65–67) impose biophysically motivated boundary conditions with respect to reaction rates in order to reduce the space of possible flux distributions. Elementary mode analysis (EMA) (68, 69) identifies all minimal subnetworks that allow a steady-state solution, thus reducing the complexity of the original network. The widely used flux balance analysis (FBA) (88) method finds a unique solution by defining an objective function to be optimized with linear programming. “Optimality” is, however, a rather subjective concept, and what optimality means for an organism is debatable (70), particularly in considering not only single organisms but also diverse communities. An extended list of examples of CBMs of microbial systems is nicely summarized in reference 89.

The typical application of CBMs is at the species level or at a higher abstraction level (Fig. 1), with the flux distribution of a single model representing the average metabolic state of a population. Examples of CBMs taking into account interspecies interactions include lumping the metabolic networks of different organisms together into a single superorganism (71) or treating different species as separate compartments, where metabolite sharing is simulated as intracompartment fluxes (72, 73). In their theoretical work, Taffs et al. (74) used EMA to study a biofilm community of sulfate-reducing bacteria, cyanobacteria, and filamentous anoxygenic phototrophs, modeled both as compartmentalized functional guilds and as a superorganism. Compared to measured data on bacterial abundances, their models offer insights into fundamental ecological questions on community composition and robustness. In particular, the identification of interguild classes of metabolic interactions associated with many elementary modes suggests the importance of such interactions to stabilization of the community.

## INTEGRATION OF MODELS, EXPERIMENTS, AND DIFFERENT SCALES

Figure 1 illustrates the key concept that the same ecosystem can be approached at different temporal and spatial scales. The integration of different methods and scales has to be theoretically justified. In dynamic FBA (dFBA) (75), for example, metabolism is considered to occur faster than external metabolite concentration changes and it is modeled with CBMs at a quasi-steady state, whereas the slower external dynamics are modeled as differential equations. dFBA has been used on compartmentalized community models (76–78), and Harcombe et al. (79) coupled these temporal dynamics with spatial discretization in a modeling framework that integrates dFBA and PDEs. Simulations of cross-feeding bacterial communities could predict cooperation and competition dynamics emerging from the spatial distribution of colonies and nutrients, followed by experimental verification.

Smriga et al. (80) studied how oligotrophic (nonmotile) and copiotrophic (motile) bacteria compete for dissolved organic matter (DOM) (organic material that is <0.7 μm in size) (90) in the phycosphere. It is estimated that up to 50% of carbon fixed via phytoplankton-mediated photosynthesis is utilized by marine bacteria (81), mainly as DOM. DOM from phytoplankton originates either from live cells or from recently lysed or grazed cells, and the quality of DOM available shapes the bacterial community in the phycosphere (82, 83). Using time-lapse microscopy, Smriga et al. measured the spatiotemporal distribution of bacteria in the phycosphere. These data were used to develop a PDE model quantifying how DOM production and consumption select for bacterial chemotaxis traits. Their results offer insights into mechanisms that can drive larger-scale ecological succession. Such a model could be, in principle, coupled to approaches like Klitgord and Segrè's (84), where CBMs are used to investigate environmental conditions driving commensalism and mutualism for different pairs of bacteria.

## CONCLUSIONS AND PERSPECTIVES

Most of our current understanding of the natural world comes from meticulous ecological and physiological studies, more recently complemented by modern high-throughput techniques such as metagenomics surveys. As this knowledge was largely driven by successful exploratory approaches, we strongly believe that it is time to develop more extensively hypothesis-driven methods to advance the field from a purely descriptive representation to a sound biological theory. This minireview presents examples of mathematical models developed to address specific biological questions related to microbial communities. We argue that modelers and experimentalists must work together from the conceptual phases of the project design to ensure correct integration of theory and experiments. In this way, a common interdisciplinary language will be developed that will aid in the unraveling of mechanisms that lie at the heart of complex natural phenomena such as microbial interactions.
